# Estimated dietary dioxin exposure and breast cancer risk among women from the French E3N prospective cohort

**DOI:** 10.1186/s13058-015-0536-9

**Published:** 2015-03-17

**Authors:** Aurélie MN Danjou, Béatrice Fervers, Marie-Christine Boutron-Ruault, Thierry Philip, Françoise Clavel-Chapelon, Laure Dossus

**Affiliations:** 1 Unité Cancer et Environnement, Centre Léon Bérard, 28 rue Laënnec, 69373 Lyon, Cedex 08 France; 2 Université Claude Bernard Lyon 1, 43 Boulevard du 11 Novembre 1918, 69100 Villeurbanne, France; 3 Inserm, Center for Research in Epidemiology and Population Health (CESP), U1018, Nutrition, Hormones and Women’s Health team, 114 rue Edouard-Vaillant, 94805 Villejuif Cedex, France; 4 Université Paris-Sud, UMRS 1018, Villejuif, France; 5 Gustave Roussy, F-94805 Villejuif, France

## Abstract

**Introduction:**

Dioxins are environmental and persistent pollutants mostly emitted from combustion facilities (e.g. waste incinerators, metal and cement industries). Known to be endocrine disrupting chemicals, dioxins are suspected to increase breast cancer (BC) risk. Although diet is considered the primary source of dioxin exposure, no previous study has been published on dietary dioxin exposure in relation to BC risk. We aimed to assess dietary dioxin exposure among women from the E3N cohort and estimate BC risk associated with this exposure.

**Methods:**

The study included 63,830 women from the E3N cohort who completed a diet history questionnaire (DHQ) in 1993 and were followed until 2008. Dietary dioxin exposure was estimated by combining consumption data from the E3N DHQ and food dioxin contamination data from a French national monitoring program. Hazard ratios (HR) and 95% confidence intervals (CI) were estimated by Cox models adjusted for BC risk factors.

**Results:**

Mean dietary dioxin exposure was estimated at 1.3 ± 0.4 pg/kg body weight (BW)/day. A 0.4 pg/kg BW/d increase in dioxin intake was not associated with overall BC risk (HR = 1.00; 95% CI: 0.96, 1.05). A significant decrease in risk of estrogen receptor negative (ER-)/progesterone receptor negative (PR-) tumors was observed among post-menopausal women in the upper quartile of estimated dioxin intake (HR for Q4 *vs*. Q1: 0.65; 95% CI: 0.45, 0.96; *P* for trend across quartiles = 0.0463).

**Conclusions:**

Overall, no association between estimated dietary dioxin exposure and BC risk was found among E3N women. Further studies should include both dietary and environmental exposures to determine whether low-dose dioxin exposure is associated with BC risk.

**Electronic supplementary material:**

The online version of this article (doi:10.1186/s13058-015-0536-9) contains supplementary material, which is available to authorized users.

## Introduction

Polychlorinated dibenzo*-p-*dioxins (PCDD) and polychlorinated dibenzofurans (PCDF), together defined as dioxins, are toxic environmental by-products resulting from the incomplete combustion of chlorinated substances from metal and cement industries, waste incinerators or domestic activities. Because they are highly lipophilic and persistent pollutants, dioxins can contaminate the food chain and accumulate in adipose tissues [1]. Therefore, in the general population, diet is considered one of the main sources of exposure to dioxins [1-3]. Most of the studies assessing dietary dioxin exposure concluded that food from animal origin rich in fat such as dairy products and meat, and seafood are the most contaminated foods. Because contribution of specific food groups to dioxin exposure depends on their consumption, main contributors might differ between populations due to different dietary patterns.

Dioxins are known to be endocrine disrupting chemicals [4] and suspected to play a role in the increase of hormone-related cancer incidence such as breast cancer. The 2,3,7,8-tetrachlorodibenzo*-p-*dioxin (TCDD) is the most toxic congener and has been classified as carcinogenic to humans by the International Agency for Research on Cancer (IARC) and the US Environmental Protection Agency (US EPA) [5,6].

Few epidemiological studies have investigated breast cancer risk associated with dioxin exposure, and there have been inconsistent results. Among the cohort of residents exposed to TCDD following the Seveso accident (Italy, 1976), no increased breast cancer risk was observed among women living in the areas most exposed to TCDD [7,8]. Nevertheless, elevated serum TCDD levels measured in Seveso women who lived in the two most contaminated zones were associated with increased breast cancer risk in a first analysis conducted in 2002 [9]. However, this was not confirmed with longer follow up of the same population [10]. Studies conducted among cohorts of workers exposed to dioxins during production of herbicides showed higher breast cancer mortality in these populations (reviewed in [11,12]). Other studies also reported elevated breast cancer mortality [13] and increased breast cancer risk [14] in cohorts of community residents exposed to dioxins emitted by industrial facilities. However in a French case-control study, a decrease in breast cancer risk was observed among older women living near a municipal solid-waste incinerator (MSWI) and most exposed to dioxins [15].

To our knowledge no epidemiological study has explored the association between dioxin exposure through diet and breast cancer risk. Therefore, our study aimed to assess dietary dioxin exposure among women from the French E3N prospective cohort and estimate breast cancer risk associated with this exposure.

## Methods

### The E3N cohort study

The E3N (*Etude Epidémiologique auprès de femmes de la Mutuelle Générale de l'Education Nationale*) study is an ongoing prospective cohort involving 98,995 French female volunteers, born between 1925 and 1950 and members of a national teachers’ health insurance plan [16]. The E3N study was initiated to identify female cancer risk factors, especially dietary, hormonal and reproductive factors. The study began in 1990 when participants returned the first questionnaire and gave signed informed consent. Since then women have completed self-administered questionnaires sent by mail every 2 to 3 years about their lifestyle, health status and medical history. The E3N study protocol was approved by the French National Commission for Data Protection and Privacy.

### Study population

Women included in our study population were participants of the E3N study, who had completed the E3N diet history questionnaire (DHQ) in 1993 and were followed until June 2008. Among the 74,522 women with dietary data, women having under- or over-reported their consumption, that is, those in the bottom and top 1% of the energy intake to basal metabolic rate ratio computed on the basis of age, height and weight, were excluded (n = 1,360). In addition, we excluded women who reported cancer diagnosis (except non-melanoma skin cancer) before completing the E3N DHQ (n = 4,705) along with those who developed *in situ* breast cancer (n = 483) and those for whom follow-up information was unavailable (n = 822). We further excluded 3,302 women with missing body weight (BW) in 1993 and 20 women who had never menstruated. Finally 63,830 women were included in our study population.

### Breast cancer cases

From the third self-administered questionnaire to the ninth, data on health status and medical history of women, including cancer occurrence, were collected and updated. A total of 3,465 women reported primary invasive breast cancer between 1993 and 2008 and 92% of them were confirmed by pathology reports. Information on estrogen receptor (ER) and progesterone receptor (PR) were extracted from these reports, and invasive breast cancer cases were classified as ER+/PR+ (n = 1,596), ER+/PR- (n = 561) or ER-/PR- (n = 414). Because of the small number of cases, the ER-/PR+ invasive breast cancers were not considered in the analyses (n = 107). Because the proportion of false-positive self-reported breast cancer in the E3N cohort was <5%, breast cancers not confirmed by pathology report (n = 285) were considered as cases. Deaths were identified through family contact or from insurance files, and causes of death were obtained from the French National Service on Causes of Death [17].

### Assessment of dietary dioxin exposure

#### Consumption data

Dietary data were collected from a validated self-administered DHQ, specifically developed for the assessment of the previous year's usual diet of the E3N population and sent in 1993 [18]. The E3N DHQ covered the daily consumption of 208 food items by collecting food frequencies and portion sizes for 8 meals and snacks during the day. The questionnaire comprised both a quantitative and a qualitative part. In the quantitative part, women were asked to indicate the frequency of consumption and to estimate portion sizes of food groups, food items and beverages. Portion sizes were estimated with a validated photo booklet [19]. The qualitative part provided detailed information on women’s relative consumption frequency of specific food items within one of the food groups of the first part. Furthermore, a food composition table was generated from a French national database [20], providing information on fat content of most of the food items assessed in the E3N DHQ.

#### Contamination data

An assessment of the level of food contamination by dioxins (PCDDs and PCDFs) in France was published in 2000 by the French High Council for Public Health (*Conseil Supérieur d’Hygiène Publique de France* (CSHPF)) [21]. These contamination data were based on food samples collected from 1996 to 1998 which was the closest period to the E3N DHQ. Food samples analyzed in this report (n = 444) included dairy products, meats, seafood products, cereal products, fruits and vegetables, eggs and fats (Additional file 1: Table S1). They were collected from monitoring and control plans mostly performed by the Directorate for food of the French Agriculture ministry (DGAL) and the General Directorate for Competition Policy, Consumer Affairs and Fraud Control (DGCCRF). Measurements of dioxin contamination in these samples were performed in one single laboratory (Carso Laboratory, Lyon, France) with a certified method using high-resolution gas chromatography/high-resolution mass spectrometry (HRGC/HRMS). Dioxin concentrations were calculated with the lower-bound method, assuming that non-detected congeners were equal to zero. Results were expressed in pg TEQ/g lipid weight, except for foods from vegetal origin (that is, cereal products and fruit and vegetables) for which results were expressed in pg TEQ/g fresh weight.

#### Exposure assessment

Dietary dioxin exposure in our study was estimated for each woman, by combining consumption data from the E3N DHQ and lipid levels from the food composition table developed by the E3N team with food dioxins contamination data from the CSHPF report (Additional file 1: Table S1), using the following formulas recommended by the World Health Organization (WHO) [22]:

Equation 1:

1 $$ {\mathrm{E}}_{\mathrm{i}}=\frac{{\displaystyle {\sum}_{\mathrm{k}=1}^{\mathrm{n}}}{\mathrm{C}}_{\mathrm{i},\mathrm{k}}\times \left({\mathrm{F}}_{\mathrm{k}}\times {\mathrm{L}}_{\mathrm{k}}\right)}{{\mathrm{BW}}_{\mathrm{i}}} $$

Equation 2, for cereal products and fruit and vegetables:

2 $$ {\mathrm{E}}_{\mathrm{i}}=\frac{{\displaystyle {\sum}_{\mathrm{k}=1}^{\mathrm{n}}}{\mathrm{C}}_{\mathrm{i},\mathrm{k}}\times {\mathrm{P}}_{\mathrm{k}}}{{\mathrm{BW}}_{\mathrm{i}}} $$

where *E*_*i*_ is the dietary dioxin exposure for the woman *i* (in pg TEQ/kg BW/day), *C*_*i,k*_ is the consumption of the food *k* by the woman *i* (in g/day), *F*_*k*_ is the proportion of lipids of the food *k* (in %), *L*_*k*_ is the average dioxin contamination of the food *k* (in pg TEQ/g lipid weight, see equation 1), *P*_*k*_ is the average dioxin contamination of the food *k* (in pg TEQ/g fresh weight, see equation 2) and *BW*_*i*_ is the body weight of the woman *i* (in kg) reported in the E3N questionnaire of 1993.

The intake of PCDDs and PCDFs was calculated in toxic equivalent (TEQ) using toxic equivalence factors (TEF) first developed by the North Atlantic Treaty Organization (NATO) in 1989 [23]. In 2001, the WHO established a dioxin-tolerable daily intake of 2.3 pg TEQ/kg BW/day [24,25].

### Statistical analysis

Participants contributed person-years of follow up from the date they completed the E3N DHQ to the date of diagnosis of primary invasive breast cancer, date of death, date of return of the ninth questionnaire or June 2008, whichever occurred first. Estimated dietary dioxin exposure was categorized according to quartiles of its distribution in the study population. Baseline characteristics of the participants were described by quartiles of estimated dietary dioxin exposure using mean and standard deviation (SD) for quantitative variables and frequency and percentage for qualitative variables.

Hazard ratios (HR) and 95% confidence intervals (CI) for breast cancer were estimated for an increase of 0.43 pg TEQ/kg BW/day (that is, 1 SD) and for quartiles of estimated dietary dioxin exposure with first quartile used as reference, using Cox proportional hazard models, stratified by 5-year-interval birth cohorts (from 1925 to 1950). Women’s age was used as the timescale. To evaluate potential dose-response associations, tests for linear trend across quartiles were derived from the Wald test of the models with the semi-continuous variable. All models were adjusted for known breast cancer risk factors and potential confounders: height (cm), body mass index (BMI) before and after menopause (cut off: 25, 30 kg/m^2^; considered as a time-dependent variable), total energy intake excluding alcohol (kcal/day), alcohol intake (cut off: 0, 6.9 g/day), education (undergraduate/postgraduate with a 1- to 4-year university degree/postgraduate with a 5+ year university degree), physical activity at baseline (cut off: 34, 46, 62 metabolic equivalent task-hour per week (MET-h/w)), smoking status (never smoked/ex-smoker/current smoker; time-dependent variable), age at menarche (cut off: 12, 14 years), previous use of oral contraceptives (never/ever), previous use of progestin before menopause (never/ever), menopausal status combined with use of menopausal hormone treatment (MHT) (premenopausal/postmenopausal non-using of MHT/postmenopausal using of MHT; time-dependent variable), age at menopause (cut off: 47, 54 years, among postmenopausal women only), age at first full-term pregnancy combined with number of live births (no child/≤2 children and age at first birth <30 years/≤2 children and age at first birth ≥30 years/≥3 children), breastfeeding (never/ever), previous family history of breast cancer (yes/no), previous history of personal benign breast disease (yes/no; time-dependent variable), previous mammography (yes/no; time-dependent variable). Further adjustments for food group consumption (including fruit and vegetable, fish and seafood, dairy products or total dietary flavonoid intake) were made. All adjustment variables had less than 5% missing data that were replaced by their modal or median value in the population. Information on estimated dietary dioxin exposure was available for all women of the study population.

We also estimated risk of invasive breast cancer according to hormone receptor status (ER/PR). We excluded cases for whom joint hormone-receptor status (n = 787) was missing from the corresponding analyses. Interactions between BMI and estimated dietary dioxin exposure were tested among premenopausal and postmenopausal women in relation to breast cancer risk because of opposite associations between BMI and risk of breast cancer before and after menopause [26] and the storage of dioxins in adipose tissues. Analyses were also stratified by weight change during follow up (cut points >−2 kg/5 years; >2 kg/5 years) and dietary patterns ("Western" *vs*. "Healthy" patterns [27]). Because of previously observed negative associations, we studied interactions between estimated dietary dioxin exposure and smoking status, and breastfeeding [28,29] while interaction between estimated dietary dioxin exposure and alcohol consumption was studied due to potential positive association [30]. All tests for interaction were derived from the likelihood-ratio test comparing the models with and without an interaction term. Sensitivity analysis was performed, excluding breast cancer cases diagnosed less than 2 years after completing the E3N DHQ, to eliminate possible preclinical tumors. All *P*-values were two-sided and the significance level was set at 0.05. We used the SAS statistical software version 9.3 (SAS Institute Inc., Cary, NC, USA) for data analysis.

## Results

We analyzed data from 63,830 women with a median follow up of 14.9 years, corresponding to 888,505 person-years and during which 3,465 incident invasive breast cancers occurred. The food groups most consumed were fruit and vegetables (mean ± SD: 724.8 ± 288.5 g/d), dairy products (341.0 ± 200.1 g/d) and cereal products (247.7 ± 108.8 g/d, Table 1). Fish and seafood were less frequently consumed (38.0 ± 27.2 g/d). The average level of dietary dioxin exposure was estimated at 1.3 ± 0.4 pg TEQ/kg BW/d (range: 0.1 to 5.7 pg TEQ/kg BW/d) and 2.7% of women exceeded the 2.3 pg TEQ/kg BW/d toxicity threshold established by the WHO [24,25]. The highest contributors to the total estimated dietary dioxin exposure were dairy products (33.3%), fruit and vegetables (22.1%), meat (18.8%) and fish and seafood (15.6%), while the contribution from eggs (5.5%), cereal products (3.6%) and added fats (1.2%) was low.

**Table 1:** **Contribution of food groups to estimated dietary dioxin exposure in the E3N cohort, 1993 (n = 63,830)**

	**Consumption (g/day),**	**Dietary dioxin exposure (pg/kg body weight/day),**	**Contribution to dietary dioxin exposure, %**
**Food groups**	**mean** ± **SD**	**mean** ± **SD**	
Dairy products	341.0 ± 417.7	0.428 ± 0.223	33.3
Fruit and vegetables	724.8 ± 288.5	0.284 ± 0.116	22.1
Meat	122.2 ± 55.2	0.242 ± 0.184	18.8
Seafood	38.0 ± 27.2	0.200 ± 0.173	15.6
Eggs	26.4 ± 20.4	0.071 ± 0.054	5.5
Cereal products	247.7 ± 108.8	0.046 ± 0.020	3.6
Added fats	25.2 ± 11.7	0.015 ± 0.013	1.2
Total	1,525.3 ± 417.7	1.285 ± 0.428	100.0

Higher estimated dietary dioxin exposure was seen in younger women and in those with higher energy intake, higher alcohol consumption, lower pre- and postmenopausal BMI, and in women with 1 or 2 children before 30 years, with a personal history of benign breast disease and those who had breastfed at least one of their children (Table 2).

**Table 2:** **Main baseline characteristics of the study population according to quartiles of estimated dietary dioxin exposure, E3N cohort, 1993 (n = 63,830)**

	**Dietary dioxin exposure (pg/kg body weight/day)**			
	**<0.98**	**[0.98, 1.23]**	**[1.23, 1.52]**	**≥1.52**
**Age (years), mean ± SD**	53.5 ± 6.8	53.0 ± 6.7	52.5 ± 6.5	51.9 ± 6.3
**Energy intake (kcal/day), mean ± SD**	1710.3 ± 391.7	2003.6 ± 405.5	2231.6 ± 437.0	2580.8 ± 513.4
**Smoking status, n (%)**				
Non-smoker	8672 (54.3)	8611 (54.0)	8614 (54.0)	8429 (52.8)
Former smoker	5102 (32.0)	5259 (33.0)	5243 (32.9)	5288 (33.1)
Smoker	2184 (13.7)	2087 (13.1)	2101 (13.2)	2240 (14.0)
**Combined menopausal status, MHT** ^**a**^ **use and BMI** ^**b**^ **, n (%)**				
Premenopausal				
BMI <25 kg/m^2^	4797 (73.7)	5665 (82.2)	6362 (87.1)	7143 (91.5)
BMI ≥25 kg/m^2^	1715 (26.3)	1227 (17.8)	942 (12.9)	667 (8.5)
Postmenopausal not using MHT				
BMI <25 kg/m^2^	2572 (58.7)	2679 (70.4)	2715 (76.5)	2692 (82.8)
BMI ≥25 kg/m^2^	1813 (41.4)	1129 (29.7)	833 (23.5)	560 (17.2)
Postmenopausal using MHT				
BMI <25 kg/m^2^	3526 (69.7)	4127 (78.5)	7261 (83.5)	4344 (88.7)
BMI ≥25 kg/m^2^	1535 (30.3)	1130 (21.5)	845 (16.6)	551 (11.3)
**Parity and age at first birth, n (%)**				
No child	2035 (12.8)	1890 (11.8)	1793 (11.2)	1685 (10.6)
≤2 children <30 years old	7661 (48.0)	7913 (49.6)	8076 (50.6)	8393 (52.6)
≤2 children ≥30 years old	1527 (9.6)	1402 (8.8)	1464 (9.2)	1378 (8.6)
≥3 children	4735 (29.7)	4752 (29.8)	4625 (29.0)	4501 (28.2)
**Breastfeeding, n (%)**				
Ever	8916 (55.9)	9234 (55.9)	9321 (58.4)	9365 (58.7)
Never	7042 (44.1)	6723 (42.1)	6637 (41.6)	6592 (41.3)
**Alcohol intake, n (%)**				
0 g/day	2636 (16.5)	2021 (12.7)	1685 (10.6)	1356 (8.5)
<6.9 g/day	6688 (41.9)	6354 (39.8)	5897 (37.0)	5181 (32.5)
≥6.9 g/day	6634 (41.6)	7582 (47.5)	8376 (52.5)	9420 (59.0)
**Personal history of benign breast disease, n (%)**				
Yes	4292 (26.9)	4570 (28.6)	4907 (30.8)	4949 (31.0)
No	11666 (73.1)	11387 (71.4)	11051 (69.3)	11008 (69.0)

^a^MHT, menopausal hormone treatment; ^b^BMI, body mass index.

Table 3 shows HRs associated with estimated dietary dioxin exposure among overall participants and according to hormone receptor status (ER/PR). An increase of 0.43 pg TEQ/kg BW/d in the intake of dioxins was not associated with risk of invasive breast cancer risk among overall participants, neither in the univariable nor in the multivariable models: HR = 1.01 (0.99, 1.03) and HR = 1.00 (0.96, 1.05), respectively. There was no statistically significant linear trend between quartiles of estimated dietary dioxin exposure and breast cancer risk (*P* for trend = 0.9405). There was also no association between estimated dietary dioxin exposure and ER+/PR+ or ER+/PR- breast cancer risk. However a borderline statistically significant decrease in ER-/PR- breast cancer risk was observed across quartiles of estimated dietary dioxin exposure (HR for Q1 *vs*. Q4: 0.72, 95% CI: 0.50, 1.02; *P* for trend across quartiles = 0.0629). When stratifying by menopausal status, we observed a statistically significant decrease in ER-/PR- breast cancer risk (HR for Q4 *vs.* Q1: 0.65, 95% CI: 0.45, 0.96; *P* for trend across quartiles = 0.0463) (Table 4).

**Table 3:** **Hazard ratios for invasive breast cancer for increased intake of 0.43 pg/kg body weight/day, according to quartiles of estimated dietary dioxin exposure and hormone receptor status (n = 63,830), 1993 to 2008**

		**Dietary dioxin exposure (pg/kg body weight/day)**				
**Populations** ^**c**^	**/0.43**	**<0.98 (ref)**	**[0.98, 1.23]**	**[1.23, 1.52]**	**≥1.52**	***P*** **-trend**
Cases, n	3465	880	853	848	884	
HR (95% CI)^a^	1.01 (0.99, 1.03)	1.00	0.97 (0.88, 1.06)	0.96 (0.88, 1.06)	1.02 (0.92, 1.11)	0.4181
HR (95% CI)^b^	1.00 (0.96, 1.05)	1.00	0.94 (0.86, 1.04)	0.93 (0.83, 1.03)	0.96 (0.85, 1.09)	0.9405
**ER+/PR+** ^**d**^						
Cases, n	1596	410	388	389	409	
HR (95% CI)^a^	1.02 (0.98, 1.07)	1.00	0.94 (0.82, 1.08)	0.95 (0.82, 1.09)	1.00 (0.88, 1.15)	0.5328
HR (95% CI)^b^	1.00 (0.93, 1.07)	1.00	0.91 (0.79, 1.06)	0.90 (0.77, 1.05)	0.94 (0.78, 1.12)	0.9678
**ER+/PR-** ^**d**^						
Cases, n	561	131	146	137	147	
HR (95% CI)^a^	1.04 (0.96, 1.13)	1.00	1.12 (0.88, 1.41)	1.05 (0.83, 1.34)	1.14 (0.90, 1.45)	0.3565
HR (95% CI)^b^	1.04 (0.93, 1.17)	1.00	1.10 (0.86, 1.41)	1.03 (0.79, 1.35)	1.14 (0.84, 1.54)	0.4571
**ER-/PR-** ^**d**^						
Cases, n	414	109	103	104	98	
HR (95% CI)^a^	0.96 (0.87, 1.06)	1.00	0.93 (0.71, 1.22)	0.94 (0.72, 1.23)	0.89 (0.67, 1.16)	0.4221
HR (95% CI)^b^	0.88 (0.77, 1.01)	1.00	0.86 (0.65, 1.13)	0.81 (0.60, 1.10)	0.72 (0.50, 1.02)	0.0629

^a^Age-adjusted models; ^b^adjusted for age, height, body mass index, energy intake, education, physical activity, smoking status, menopausal status combined with use of menopausal hormone treatment, alcohol intake, age at menarche, use of oral contraceptives, use of progestin, age at menopause, age at first full-term pregnancy and number of live births, breastfeeding, family history of breast cancer, history of personal benign breast disease and mammography; ^c^ER-/PR+ invasive breast cancer risk was not analyzed due to small number of cases (n = 107); ^d^n = 787 invasive breast cancer cases with missing hormone receptor status. HR, hazard ratio.

**Table 4:** **Hazard ratios for pre- and postmenopausal breast cancer risk for increased intake of 0.43 pg/kg body weight/day, according to quartiles of estimated dietary dioxin exposure (n = 63,830), 1993 to 2008**

		**Dietary dioxin exposure (pg/kg body weight/day)**				
**Populations**	**/0.43**	**<0.98 (ref)**	**[0.98, 1.23]**	**[1.23, 1.52]**	**≥1.52**	***P*** **-trend**
**Premenopausal women** ^**c**^						
Cases, n	446	104	105	117	120	
HR (95% CI)^a^	1.03 (0.94, 1.13)	1.00	0.93 (0.71, 1.22)	0.96 (0.74, 1.25)	0.94 (0.73, 1.23)	0.5705
HR (95% CI)^b^	0.98 (0.87, 1.12)	1.00	0.84 (0.63, 1.11)	0.83 (0.62, 1.11)	0.77 (0.54, 1.08)	0.7873
**Postmenopausal women** ^**d**^						
Cases, n	3019	775	749	731	764	
HR (95% CI)^a^	1.01 (0.98, 1.05)	1.00	0.97 (0.88, 1.08)	0.96 (0.87, 1.06)	1.03 (0.93, 1.14)	0.5134
HR (95% CI)^b^	0.98 (0.93, 1.03)	1.00	0.94 (0.84, 1.04)	0.90 (0.80, 1.01)	0.93 (0.82, 1.06)	0.3827
**ER+/PR+** ^**e**^						
Cases, n	1365	354	336	325	350	
HR (95% CI)^a^	1.02 (0.96, 1.07)	1.00	0.96 (0.82, 1.11)	0.94 (0.81, 1.09)	1.03 (0.89, 1.20)	0.5837
HR (95% CI)^b^	0.97 (0.90, 1.04)	1.00	0.91 (0.78, 1.06)	0.86 (0.73, 1.02)	0.91 (0.75, 1.10)	0.4193
**ER+/PR-** ^**e**^						
N cases	513	117	136	125	135	
HR (95% CI)^a^	1.05 (0.96, 1.14)	1.00	1.17 (0.92, 1.50)	1.09 (0.85, 1.40)	1.20 (0.93, 1.53)	0.2780
HR (95% CI)^b^	1.05 (0.93, 1.18)	1.00	1.16 (0.89, 1.49)	1.07 (0.81, 1.41)	1.19 (0.86, 1.63)	0.4341
**ER-/PR-** ^**e**^						
N cases	359	98	91	89	81	
HR (95% CI)^a^	0.95 (0.85, 1.06)	1.00	0.93 (0.70, 1.23)	0.91 (0.68, 1.21)	0.84 (0.62, 1.13)	0.3261
HR (95% CI)^b^	0.86 (0.74, 1.00)	1.00	0.84 (0.63, 1.13)	0.77 (0.56, 1.07)	0.65 (0.45, 0.96)	0.0463

^a^Age-adjusted models; ^b^adjusted for age, height, body mass index, energy intake, education, physical activity, smoking status, menopausal status combined with use of menopausal hormone treatment, alcohol intake, age at menarche, use of oral contraceptives, use of progestin, age at menopause, age at first full-term pregnancy and number of live births, breastfeeding, family history of breast cancer, history of personal benign breast disease and mammography; ^c^results for premenopausal breast cancer risk according to hormone receptor status were not presented due to the small number of cases; ^d^ER-/PR+ postmenopausal invasive breast cancer risk was not analyzed due to small number of cases (n = 73); ^e^n = 709 postmenopausal invasive breast cancer cases with missing hormone receptor status. HR, hazard ratio.

Adjusting for fruit and vegetable consumption, total dietary flavonoid intake, or any other food groups, did not change the results (data not shown). There was no significant interaction between BMI and estimated dietary dioxin exposure among pre- and postmenopausal women in relation to invasive breast cancer risk. There was no effect modification by smoking, alcohol consumption, dietary patterns, gain or loss of weight during follow up and breastfeeding, and there was no association between estimated dietary dioxin exposure and breast cancer risk in any above-defined subgroups (Additional file 1: Table S2). Our results did not change when excluding breast cancer cases diagnosed less than 2 years after inclusion (data not shown).

## Discussion

To our knowledge, this study is the first to assess breast cancer risk associated with dietary dioxin exposure. Among women from the E3N prospective cohort, we did not observe any increase of breast cancer risk associated with estimated dietary dioxin exposure. Among postmenopausal women, an inverse association was observed between estimated dioxin intake and ER-/PR- breast cancer risk.

Our estimation of an average dioxin intake of 1.3 pg TEQ/kg BW/d is of the same order of magnitude or lower than in other studies. In a monitoring program conducted in 1996 to 1998 in the general French population and using the same dioxin contamination data as in our study, mean dietary dioxin exposure was also estimated at 1.3 pg TEQ/kg BW/d [21]. At the same period in Europe, estimated dietary dioxin exposure of the general population varied between 1.0 pg TEQ/kg BW/d (Germany) and 3.5 pg TEQ/kg BW/d (Spain) [31-34]. In the US, dietary dioxin exposure was estimated between 0.3 and 3.2 pg TEQ/kg BW/d [35]. Dairy products and seafood were consistently identified across studies as being the main food contributors to dioxin exposure [21,32,34]. However, because food contribution depends on dietary habits, some studies (including ours) showed an additional important contribution of cereal products and fruit and vegetables [31]. These foods, although little contaminated by dioxins, are highly consumed by some populations, and their contribution to overall dioxin intake must thus be considered. However, most previous studies focused on dioxin intake from foods known to be the most contaminated by dioxins and did not consider dioxin intake from consumption of foods from vegetal origin. This is likely to result in an underestimation of the intake of dioxins, as concluded in a validation study of a dietary questionnaire developed for the assessment of dietary dioxin exposure [36].

Previous studies investigating the association between breast cancer and dioxin exposure (environmental, accidental and occupational) mostly reported a weak increase in the risk of breast cancer [9,14] or the mortality rates of breast cancer [11-13]. Our results are not consistent with those studies, probably because they have focused on populations exposed accidentally, occupationally or living near emitting sources of dioxins, that is, exposed to high levels of dioxins, whereas our study population was exposed to low levels of dioxins. Moreover, among the Seveso Women's Health Study (SWHS) [9], the results were obtained for a different exposure window than women from the E3N study, as the SWHS women were aged 0 to 40 at the time of exposure (1976) whereas in 1993, almost half of the women of our study population were postmenopausal.

Our results suggest that exposure to dioxins in the low range observed in our study may be associated with a decreased risk of hormone-independent (ER-/PR-) breast cancer, especially after menopause. Inverse associations between endocrine disrupting chemicals and ER- breast cancer risk have been found previously. Raaschou-Nielsen *et al.* found a lower risk associated with higher adipose tissue concentrations of dioxin-like organochlorines in postmenopausal women [37]. Gammon *et al.* observed a lower risk of ER-/PR- breast cancer in women with serum levels of PCBs in the highest tertile compared to the lowest [38]. Some studies investigating breast cancer risk in relation to dioxin exposure also suggested a decreased breast cancer risk. A French registry-based case-control study conducted near a MSWI showed a decreased risk of invasive breast cancer among women aged over 60 years and highly exposed, compared to non-exposed women, although these results could be explained by confounding, because there was no adjustment on breast cancer risk factors or other potential confounders other than age and place of residence [15]. A small decrease in breast cancer risk was also observed among women exposed during the Seveso accident and followed between 1977 and 1986, although not statistically significant [7]. However, the authors of these studies did not stratify on tumor hormone receptor status. Our findings may also be supported by experimental evidence on the role of dioxins in promotion or inhibition of mammary tumor formation. TCDD has been shown to operate in animals and humans through binding to the aryl hydrocarbon receptor (AhR, [5]). Some studies have described an antiproliferative action of TCDD in ER- breast cancer cell lines through AhR-dependent [39,40] or AhR-independent pathways [41]. These observations were complemented by animal studies demonstrating that TCDD inhibits the proliferation of cells from the mammary gland and mammary tumor formation [42,43]. In a recent review of the literature on the role of AhR in carcinogenesis, Safe *et al.* highlighted the fact that the effect of TCDD may vary according to the target organ as well as windows of exposure [44]. For example, animal studies have demonstrated that prenatal TCDD exposure increased susceptibility to carcinogen-induced mammary tumor formation, while exposure during pregnancy delayed mammary tumor formation. Women of our study population were on average aged 57.2 ± 6.6 years old at inclusion in 1993. Therefore, we were not able to investigate the association of breast cancer risk with estimated dietary dioxin exposure during other windows of exposure such as prenatal periods or pregnancy.

### Strengths and limitations

Strengths of our study include the prospective design, the large size of the study population and number of breast cancer cases, a long follow-up that provides a large latency period for potential cancer occurrence, adjustment for most known breast cancer risk factors updated throughout follow-up, and validated dietary data. The participation rate which is still high 18 years after the start of the E3N study (≥75% at each follow-up questionnaire) and the low percentage of missing data attests of the quality of the information collected. Because our statistical models were adjusted for several individual breast cancer risk factors, it is unlikely that residual confounding changes our risk estimates. Moreover, we analyzed the potential confounding effect of flavonoid intake. Flavonoids are dietary phytochemicals that have been suggested to protect against dioxin toxicity [45] and to play a role in breast cancer prevention [46,47]; adjustment for total dietary flavonoid intake did not change our results.

Nevertheless, our study has some limitations. First, to determine dietary dioxin exposure, we used consumption data obtained through a semi-quantitative diet history questionnaire. DHQ are often used for their applicability to a large number of participants. However, as DHQ are self-administered questionnaires on habitual food consumption over the past 12 months, we cannot exclude some inaccuracy of consumption data and a non-differential bias due to the desire of conformity to social norms. Nevertheless, the potential lack of precision has been minimized by the collection of food frequency for each food item and the estimation of portion sizes through a photo-booklet. Moreover, both the dietary questionnaire and photo-booklet have been validated [18,19].

Second, the food contamination data by dioxins came from the first French national monitoring program, which aimed to assess dioxin levels of foods sampled between 1996 and 1998 [21]. Because the authors did not achieve an exhaustive sampling plan, the data may not be representative of the dioxin contamination of food in France for that period. However, because of the large number of samples (n = 444), the coverage of all major food contributors of dioxin intake and the fact that those data were the closest to the period of the E3N DHQ administration, we can assume that they accurately reflect the contamination of food in 1993. We combined the consumption data with the contamination data using standardized formulas recommended by the WHO [22]. However, for some food items of the E3N DHQ, no contamination data were reported in the CSHPF study; therefore we assigned to each of these items the average contamination of the food group to which it belonged (see Additional file 1: Table S1 for an example).

Also, we could not consider the source of the food consumed, that is, women living and consuming food produced near a dioxin emitting source may consume foods more contaminated than average. This could have led to an underestimation of the exposure for those women and to misclassification bias, although non differential so the risk would be potentially drawn toward 1. The estimated dietary dioxin exposure may not reflect the total exposure to dioxins of the E3N women. We cannot eliminate the contribution of exposure from environmental sources, even if its contribution remains probably minor compared to that of food. In contrast, because most women from the E3N cohort are teachers or from affiliated occupations, we can assume the occupational dioxin exposure to be negligible and homogeneous among study participants.

Our estimation of the dietary dioxin exposure cannot be extrapolated to the French general population. Indeed, the intake of dioxins through the diet depends on the relative intake of foods of high or low levels of dioxin contamination and their quantity consumed (for instance, our population has a higher consumption of fruit and vegetables than generally observed). Moreover, we can expect a healthy participation effect in our cohort, as it is composed of volunteers with a high level of education and health consciousness. This could result in a possible underrepresentation of high fat (so high dioxin) consumptions; however this should not have biased our results.

## Conclusion

Among women from the E3N prospective cohort, estimated dietary dioxin exposure was not associated with breast cancer risk. Further studies should include both dietary and non-dietary (environmental) exposures as well as different windows of exposure (prenatal, pregnancy periods) in order to further investigate the complex association between low-dose dioxin exposure and hormone receptor-defined breast cancer risk.

## Additional file

Additional file 1: Table S1. Dioxin contamination and lipids proportion of food items from the E3N diet history questionnaire of 1993. **Table S2.** Hazards ratios (HR) for invasive breast cancer in subgroups defined by body mass index, weight change, dietary patterns and breastfeeding (n = 63,830), 1993 to 2008.

## Abbreviations

AhR: aryl hydrocarbon receptor
BMI: body mass index
BW: body weight
CSHPF: *Conseil Supérieur d’Hygiène Publique de France*
DHQ: diet history questionnaire
E3N: *Étude Épidémiologique auprès de femmes de la Mutuelle Générale de l’Education Nationale*
ER: estrogen receptor
HR: hazard ratio
MSWI: municipal solid-waste incinerator
PCDD: polychlorinated dibenzo*-p-*dioxin
PCDF: polychlorinated dibenzofuran
PR: Progesterone receptor
TCDD: 2,3,7,8-tetrachlorodibenzo*-p-*dioxin
TEQ: toxic equivalency
WHO: World Health Organization
