# Supplementary material for: Estimated dietary dioxin exposure and breast cancer risk among women from the French E3N prospective cohort
Source: Breast Cancer Res. 2015 Mar 17;17:39. doi: 10.1186/s13058-015-0536-9 (PMC4362830; doi:10.1186/s13058-015-0536-9)
Supplement: Additional file 1: Table S1. — Dioxin contamination and lipids proportion of food items from the E3N diet history questionnaire of 1993. Table S2. Hazards ratios (HR) for invasive breast cancer in subgroups defined by body mass index, weight change, dietary patterns and breastfeeding (n = 63,830), 1993 to 2008. [file 13058_2015_536_MOESM1_ESM.pdf]

Additional File 1

Table S1. Dioxin contamination and lipids proportion of food items from the E3N diet history questionnaire of 1993.

**Food groups**	**Food items**	**Dioxin contamination level (pg I-TEQ/g lipid weight or *fresh weight*)[a]**	**Lipids proportion (%)[c]**
**Meat**	Beef	0.80	8.86
	Horse	0.60[b]	4.60
	Lamb	0.74	14.09
	Calf	0.48	5.12
	Pork	0.16	11.04
	Offal	3.32	15.42
	Charcuterie	0.25	16.41
	Poultry	0.60	6.76
	Rabbit	0.60[b]	8.70
**Cereal products**	Pasta	*0.01*	-
	Rice	*0.01*	-
	Cereals	*0.02*	-
	Bread	*0.01*	-
	Biscuits/cakes	*0.0125*	-
**Eggs**	Eggs	1.51	10.50
**Fish and Seafood**	Trout	9.36	4.20
	Sardines	2.70	12.30
	Colin/hake	2.24	1.00
	Ling	4.64[b]	1.00
	Mackerel	3.23	20.90
	Flounder	4.64[b]	1.00
	Haddock	4.64[b]	1.00
	Sole	4.64[b]	1.00
	Saithe	3.38	1.00
	Whiting	34.16	0.90
	Cod	8.45	0.95
	Salmon	5.15	10.78
	Saumonette	4.64[b]	16.30
	Other fish	5.13	4.18
	Canned fish	4.64[b]	8.50
	Seafood	30.22	2.77
**Dairy products**	Milk	0.65	2.32
	Butter	0.92	68.67
	Pressed cheese	0.64	29.25
	Other cheese	0.77	30.10
	Cream	0.68	27.00
	Fromage blanc	1.16	3.35

	Dairy dessert	1.16	5.04
	Yogurt	1.16	1.80
**Fruit and vegetables**	Fruits	*0.01*	-
	Leafy vegetables	*0.055*	-
	Root vegetables	*0.01*	-
	Other vegetables	*0.032*	-
	Soup	*0.034*[b]	-
**Added fats**	Animal fats	0.88	99.75
	Peanut oil	0.03	83.25
	Olive oil	0.04	83.25
	Margarine	0.04	61.99
	Sunflower oil	0.04[b]	83.25
	Corn oil	0.04[b]	83.25
	Other vegetable fats	0.04[b]	70.75
	Mayonnaise	0.04[b]	78.60

[a] Dioxin contamination data of 1996-1998 provided by the French High Council for Public Health (Conseil Supérieur d'Hygiène Publique de France).

[b] The average dioxin contamination of the food group was assigned to the food item.

[c] Lipids proportion provided by the food composition table developed by the E3N team.

Table S2. Hazards ratios (HR) for invasive breast cancer in subgroups defined by body mass index, weight change, dietary patterns and breastfeeding (N=63,830), 1993-2008.

	Dietary dioxin exposure (pg/kg body weight/day)					
**Stratifications**	**/0.43**	**<0.98 (ref)**	**[0.98,1.23[**	**[1.23,1.52[**	**≥1.52**	**p-trend**
**Body Mass Index (BMI)**						
**BMI<25 kg/m²**						
N cases	2778	600	684	706	788	
HR (95% CI)[a]	1.01 (0.97, 1.05)	1.00	0.99 (0.89, 1.11)	0.96 (0.86, 1.07)	1.01 (0.91, 1.13)	0.5931
HR (95% CI)[b]	0.98 (0.93, 1.03)	1.00	0.96 (0.86, 1.07)	0.90 (0.80, 1.02)	0.93 (0.81, 1.06)	0.4682
**BMI≥25 kg/m²**						
N cases	687	280	169	142	96	
HR (95% CI)[a]	1.01 (0.93, 1.10)	1.00	0.87 (0.72, 1.06)	0.99 (0.81, 1.21)	0.99 (0.78, 1.25)	0.7633
HR (95% CI)[b]	1.00 (0.89, 1.12)	1.00	0.85 (0.69, 1.04)	0.94 (0.75, 1.18)	0.94 (0.70, 1.26)	0.9875
**Weight change[c]**						
**Weight loss (>-2 kg/5 years)**						
N cases	211	56	59	47	49	
HR (95% CI)[a]	1.03 (0.91, 1.18)	1.00	1.19 (0.83, 1.72)	1.06 (0.72, 1.57)	1.12 (0.77, 1.65)	0.6288
HR (95% CI)[b]	1.00 (0.84, 1.19)	1.00	1.11 (0.76, 1.63)	1.00 (0.65, 1.54)	1.01 (0.62, 1.64)	0.9796
**Stable weight ([-2;2] kg/5 years)**						
N cases	2014	501	482	498	533	
HR (95% CI)[a]	1.00 (0.95, 1.04)	1.00	0.90 (0.80, 1.02)	0.89 (0.79, 1.01)	0.95 (0.84, 1.07)	0.9628
HR (95% CI)[b]	1.00 (0.94, 1.07)	1.00	0.89 (0.78, 1.01)	0.88 (0.76, 1.01)	0.92 (0.79, 1.08)	0.9213
**Weight gain (>2 kg/5 years)**						
N cases	1234	320	310	303	301	
HR (95% CI)[a]	1.04 (0.99, 1.10)	1.00	1.04 (0.89, 1.22)	1.09 (0.93, 1.27)	1.13 (0.96, 1.32)	0.1350
HR (95% CI)[b]	1.00 (0.93, 1.08)	1.00	1.00 (0.85, 1.18)	1.00 (0.84, 1.19)	1.02 (0.83, 1.25)	0.9893
**Western Pattern**						
**Low intake**						
N cases	1059	483	299	198	79	
HR (95% CI)[a]	0.99 (0.91, 1.07)	1.00	1.03 (0.89, 1.19)	1.12 (0.95, 1.33)	0.96 (0.75, 1.22)	0.7361
HR (95% CI)[b]	0.98 (0.88, 1.08)	1.00	1.03 (0.89, 1.20)	1.12 (0.94, 1.34)	0.97 (0.76, 1.24)	0.6586
**Medium intake**						

N cases	1163	274	321	349	219	
HR (95% CI)[a]	0.96 (0.89, 1.03)	1.00	0.87 (0.74, 1.02)	0.95 (0.81, 1.11)	0.81 (0.67, 0.96)	0.2150
HR (95% CI)[b]	0.94 (0.86, 1.02)	1.00	0.85 (0.73, 1.01)	0.93 (0.79, 1.09)	0.78 (0.65, 0.95)	0.1438
**High intake**						
N cases	1243	110	222	386	525	
HR (95% CI)[a]	0.98 (0.93, 1.03)	1.00	0.92 (0.73, 1.15)	0.94 (0.76, 1.16)	0.85 (0.69, 1.05)	0.4446
HR (95% CI)[b]	1.02 (0.96, 1.09)	1.00	0.94 (0.74, 1.18)	0.98 (0.78, 1.21)	0.89 (0.72, 1.11)	0.4666
**Healthy pattern**						
**Low intake**						
N cases	1166	375	292	287	212	
HR (95% CI)[a]	1.01 (0.94, 1.08)	1.00	0.96 (0.82, 1.12)	1.06 (0.91, 1.24)	0.88 (0.74, 1.05)	0.8799
HR (95% CI)[b]	1.00 (0.91, 1.10)	1.00	0.93 (0.80, 1.09)	1.02 (0.86, 1.20)	0.83 (0.69, 1.01)	0.9751
**Medium intake**						
N cases	1164	290	292	319	263	
HR (95% CI)[a]	0.98 (0.92, 1.05)	1.00	1.00 (0.85, 1.18)	1.05 (0.90, 1.23)	0.92 (0.78, 1.10)	0.6134
HR (95% CI)[b]	1.00 (0.91, 1.09)	1.00	1.00 (0.85, 1.18)	1.04 (0.87, 1.23)	0.90 (0.74, 1.09)	0.9096
**High intake**						
N cases	1135	202	258	327	348	
HR (95% CI)[a]	1.06 (1.01, 1.12)	1.00	1.08 (0.90, 1.30)	1.24 (1.04, 1.48)	1.23 (1.03, 1.46)	0.0248
HR (95% CI)[b]	1.05 (0.98, 1.13)	1.00	1.06 (0.88, 1.28)	1.21 (1.00, 1.46)	1.17 (0.95, 1.43)	0.1574
**Breastfeeding**						
**No breastfeeding**						
N cases	1483	382	366	371	364	
HR (95% CI)[a]	1.02 (0.97, 1.07)	1.00	1.00 (0.87, 1.15)	1.03 (0.89, 1.19)	1.02 (0.88, 1.18)	0.4492
HR (95% CI)[b]	1.02 (0.95, 1.09)	1.00	0.97 (0.84, 1.13)	0.99 (0.84, 1.16)	0.99 (0.82, 1.19)	0.6577
**Breastfeeding**						
N cases	1982	497	488	477	520	
HR (95% CI)[a]	1.01 (0.97, 1.06)	1.00	0.95 (0.84, 1.07)	0.92 (0.81, 1.04)	1.01 (0.89, 1.14)	0.6589
HR (95% CI)[b]	0.99 (0.93, 1.05)	1.00	0.92 (0.81, 1.05)	0.88 (0.76, 1.01)	0.94 (0.80, 1.11)	0.7427
**Smoking Status**						
**Non-smoker**						
N cases	1861	492	469	431	469	
HR (95% CI)[a]	1.00 (0.96, 1.05)	1.00	0.96 (0.84, 1.09)	0.88 (0.77, 1.00)	0.99 (0.87, 1.12)	0.9040
HR (95% CI)[b]	0.99 (0.92, 1.05)	1.00	0.93 (0.81, 1.06)	0.83 (0.72, 0.96)	0.92 (0.78, 1.09)	0.6414
**Former smoker**						
N cases	1158	278	273	309	298	
HR (95% CI)[a]	1.03 (0.97, 1.09)	1.00	0.95 (0.81, 1.13)	1.09 (0.92, 1.28)	1.05 (0.89, 1.23)	0.2918

HR (95% CI)[b]	1.03 (0.95, 1.11)	1.00	0.94 (0.79, 1.12)	1.07 (0.89, 1.28)	1.03 (0.84, 1.27)	0.4625
**Smoker**						
N cases	446	109	112	108	117	
HR (95% CI)[a]	1.01 (0.93, 1.11)	1.00	1.07 (0.82, 1.39)	1.03 (0.79, 1.34)	1.05 (0.81, 1.36)	0.7549
HR (95% CI)[b]	1.00 (0.88, 1.13)	1.00	1.03 (0.78, 1.36)	0.97 (0.72, 1.30)	0.98 (0.70, 1.37)	0.9852
**Alcohol intake**						
**0 g/day**						
N cases	375	134	90	68	83	
HR (95% CI)[a]	1.08 (0.98, 1.19)	1.00	0.86 (0.66, 1.13)	0.78 (0.58, 1.05)	1.21 (0.92, 1.59)	0.1448
HR (95% CI)[b]	1.02 (0.89, 1.17)	1.00	0.79 (0.60, 1.04)	0.68 (0.49, 0.94)	0.99 (0.69, 1.42)	0.7708
**<6.9 g/day**						
N cases	1234	348	338	294	254	
HR (95% CI)[a]	0.99 (0.93, 1.05)	1.00	1.02 (0.88, 1.19)	0.96 (0.82, 1.12)	0.96 (0.81, 1.12)	0.6697
HR (95% CI)[b]	1.00 (0.92, 1.08)	1.00	1.03 (0.88, 1.20)	0.96 (0.81, 1.14)	0.96 (0.78, 1.19)	0.9285
**≥6.9 g/day**						
N cases	1856	397	426	486	547	
HR (95% CI)[a]	1.00 (0.96, 1.05)	1.00	0.94 (0.82, 1.07)	0.96 (0.84, 1.10)	0.97 (0.86, 1.11)	1.0000
HR (95% CI)[b]	1.01 (0.95, 1.07)	1.00	0.92 (0.80, 1.06)	0.95 (0.82, 1.11)	0.97 (0.82, 1.15)	0.8817

[a] age-adjusted models.

[b] adjusted for age, height, body mass index, energy intake, education, physical activity, smoking status combined with use of menopausal hormone treatment, alcohol intake, age at menarche, use of oral contraceptives, use of progestin, age at menopause, age at first full-term pregnancy and number of live births, breastfeeding, family history of breast cancer, history of personal benign breast disease and mammography.

[c] n=6 breast cancer cases with missing data for weight change during follow-up.
